# A discussion on implementing pooling detection tests of novel coronavirus (SARS-CoV-2) for a large population

**DOI:** 10.1017/S0950268820003155

**Published:** 2021-01-05

**Authors:** W. K. Chow, C. L. Chow

**Affiliations:** 1 Department of Building Services Engineering, The Hong Kong Polytechnic University, Hong Kong, China; 2 Department of Architecture and Civil Engineering, City University of Hong Kong, Hong Kong, China

**Keywords:** Coronavirus, COVID-19, laboratory tests, mathematical modelling

## Abstract

A pooled sample analysis strategy for novel coronavirus (severe acute respiratory syndrome-coronavirus-2 (SARS-CoV-2)) is proposed for a large population in this paper. The population to be tested is divided into divisions based on earlier observed detection rate of SARS-CoV-2 first. Samples collected are then grouped in appropriate pooled size. The number of tests per person in that population is expressed as a function of two variables: the observed detection rate and the pooled size or number of samples grouped. The minimum number of tests per person can be further shown to be a function of only one of these two variables, because these two parameters are found to be related at this minimum. A management scheme on grouping the samples is proposed in order to reduce the number of tests, to save time, which is of utmost importance in fighting an epidemic. The proposed testing scheme will be useful for supporting the government in making decisions to handle regular routine detection tests for identifying asymptomatic patients and implementing health code system in large population of millions of citizens. Another important point is to use smaller number of test kits, allowing more resources to speed up the mass screening tests, particularly in places not so rich.

## Introduction

Spreading of the novel coronavirus (severe acute respiratory syndrome-coronavirus-2 (SARS-CoV-2)) and the related disease coronavirus disease 2019 (COVID-19) among people is very fast [1]. The origin of such virus is unknown with the transmission route not yet fully understood, though some physical models have been proposed [2, 3] to explain the virus characteristics. There were only 0.75 million confirmed cases in the end of March, 25 million in the end of August, 50 million in the beginning of November and over 70 million in December 2020 all over the world [1]. Lockdown measures were imposed [4] to slow its spread. Most countries have to close their borders with appropriate quarantine containment schemes [5, 6] for weeks, affecting normal life and business activities. The collapse [7] of global health systems caused by COVID-19 could unleash synergistic public health crises (e.g. cholera) caused by both known and unknown opportunistic pathogens.

Millions of detection tests are needed to confirm infection and pick out asymptomatic patients (AP) for each wave of infection, practising universal testing and implementing health codes [8] in large populations. A data-driven approach reveals three key strategies in tackling COVID-19 by Lai and Cheong [9]. The scale of testing has to be suggested by health experts to contain the virus for better effect. Slow testing due to spending a long time on queueing means that AP continue to spread the virus in the community, making the battle against COVID-19 more difficult because of the hidden carriers. A large number of detection tests are required regularly for safely keeping economic activities, travelling, normal school activities and others, particularly on small and medium-sized enterprises (SME). High damages to SME are observed all over the world because the owners have to pay the rent, mortgage, staff salary and other expenses even when the shops are closed. Airlines are closed with aeroplanes parking at dry deserts. Globalisation is seriously affected under such conditions. Different views on the impact of different waves of infection [4, 7] were also reported. Quick identification tests for the SARS-CoV-2 virus is needed regularly for millions of people.

However, a test will normally take over 6 h to complete. The associated testing cost and the cost of the test kit are high, about US$3 to US$5 in Mainland China but over US$30 [10] in many other countries. There are difficulties in getting adequate number of test kits in some countries. The maximum number of tests the government can handle in many places is limited, say only 10 000 a day in the Hong Kong Special Administrative Region (HKSAR) with a population of 7 million in the end of July. With great effort made by the HKSAR government, the number of tests increased to over 30 000 per day in August. This was to be increased to a minimum of 100 000 per day in September with strong support from Mainland China [11].

Pooling tests were reported years ago [12] with application to COVID-19 recently [13–16]. Such testing methodology is used in many places [17] by combining the samples to save time (particularly the queueing time) and resources. Different pool sizes [13, 15, 16] are summarised as shown in Table 1.

**Table 1.** Brief summary on pool size used in the literature

References	Pool sample
Pikovski and Bentele [13]	3, 4 and 5
Yelin *et al*. [15]	32 and 64
Deckert *et al*. [16]	3−25, can be up to 27
Adikari *et al*. [20]	32
Elsevier [27]	10
Majid *et al*. [28]	Up to 64
Mishra *et al*. [29]	30

Optimal parameters for group testing of pooled specimens for the detection of SARS-CoV-2 were established recently [18]. The most efficient pool size was determined to be five specimens using a web-based application. This is appropriate [19] for testing asymptomatic and return-to-work/school cases, where the prevalence of COVID-19 may be relatively low, large groups of clinical samples can be classified as negative with a single test, with no need to test every sample individually. Group testing can save reagents and personnel time with an overall increase in testing capability of at least 69%. Different pooling strategies [20] can lead to increased process efficiencies for COVID-19 clinical diagnostic testing.

Mathematical strategy of such pooling tests applied to COVID-19 was briefly outlined [14, 21]. There were some criticisms on using the proposal [22] in the HKSAR in reducing the testing time. In fact, quick, reliable and cheap detection testing schemes with smaller number of tests can only be worked out by appropriate safety management scheme. Further works are needed to evaluate whether such mathematical strategy [14, 21] is viable.

Randomised group testing optimised per country could double the number of tested individuals from 1.85 million to 3.7 million using only 671 000 tests [23]. Pooling approaches for SARS-CoV-2 testing allow a drastic increase in throughput while maintaining clinical sensitivity. Successful large-scale pooled screening of asymptomatic populations [24] was reported. As a clinic or hospital is unable to handle a large number of tests, special testing centres are established for testing COVID-19 in many places including Hong Kong.

In this paper, the population to be tested is proposed to be divided into divisions based on the observed recent detection rate of SARS-CoV-2. The number of tests per person for such pooling detection tests can be expressed as a function of two parameters. The parameters are the observed detection rate of getting one positive result in testing a group of *m* people, and the pooled size or number of samples *n* in putting together for a pool test in that group of collected samples. The mathematical aspects [25, 26] of the function are discussed in the following sections. A management scheme can be worked out to reduce the number of tests so as to consume a smaller number of test kits and shorten the queuing time so that citizens need not wait for several days to get results. This approach of pooling tests is very appropriate in practising health code system [8] in handling large number of tests regularly.

## Mathematical analysis

A population of *P* people to be tested is divided into (*P*/*m*) divisions each of *m* people first. The total number of tests in this population with a group testing scheme is *L*. This gives the average number of tests per person, to be expressed as a function *f*(*m*, *n*) of two variables:1
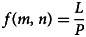


The two variables are:

- *m*, the average number of people per 1 positive detection in recent test; and
- *n*, pooled size or number of samples grouped together for a pool test.

Samples collected from each person in each division are grouped by *n* persons for pool testing. There will be about *y*_*i*_ positive results in a division *i*.

In the *j*th division, the number of positive tests *y*_*j*_ is most probably 1. However, there might be some divisions having 0, 2 or 3 in a large population *P.* The average value of *y*_*j*_ is 1. This is because the infected patients are not uniformly distributed in each division. If the government implemented better quarantine containment scheme [5, 6], *m* can be increased, say instead of having 1 positive result in 100 tests, to 1 positive detection testing in 1000 people. That is, *m* can be increased from 100 to 1000.

The number of tests *L*_*j*_ in that *j*th division [26] is2
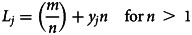


Summing over all divisions for a large sample of *P*, say 1 million, with *j* from 1 to *P*/*m* gives3
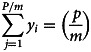


Summing over all *L*_*j*_ in equation (2) will give *L* as
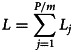


or4
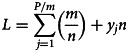


by putting equation (3) into equation (4) will give *L*.

Putting equation (4) into equation (1) gives *f*(*m*, *n*) as
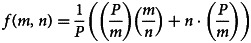
or5
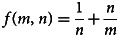


**Fig. 1.**
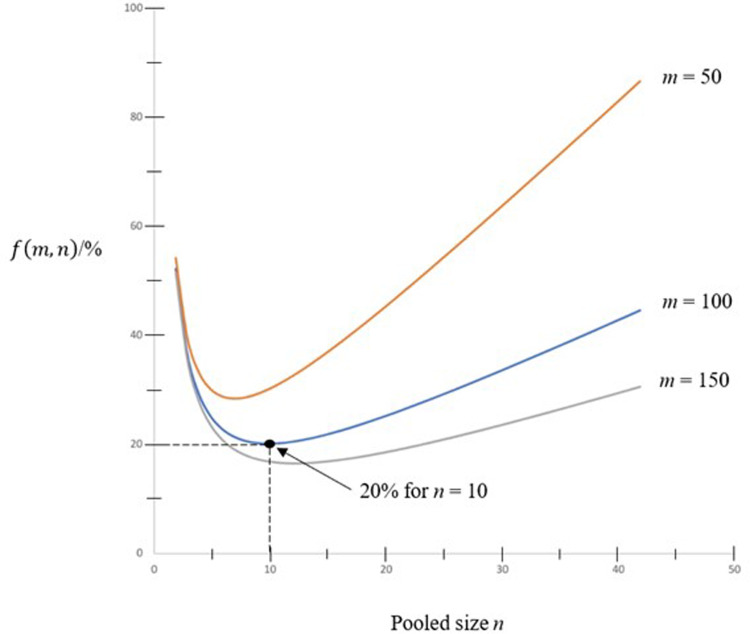
Curves of ***f***(***m***, ***n***) against pooled size ***n***.

Curves of *f*(*m*, *n*) against the pooled sized *n* with some values of *m* = 50, 100 and 150 are shown in Figure 1. As shown in the figure, the minimum value of *f*(*m*, *n*) for *m* = 100 is 0.2 when *n* = 10. That means by pooling 10 samples together, only 20% of tests are required in comparing to doing one test for each person in the population.

**Fig. 2.**
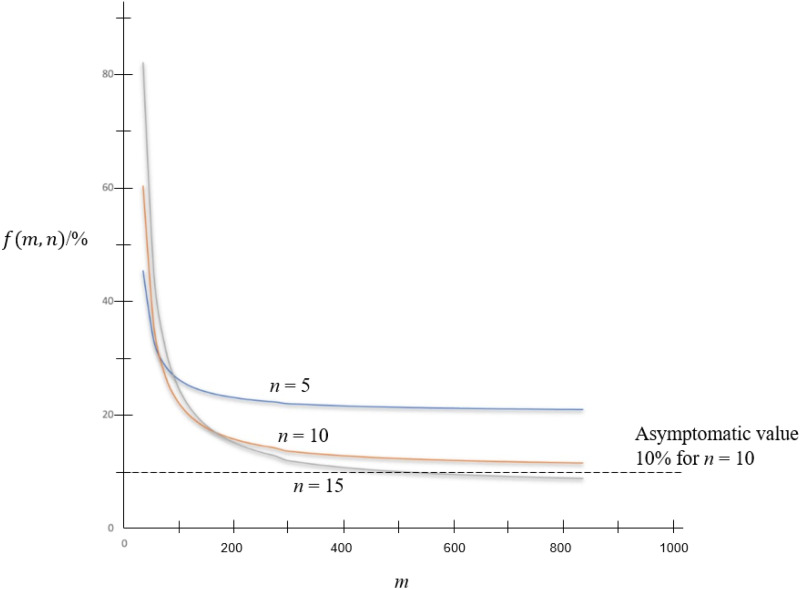
***f***(***m***, ***n***) against ***m***.

In keeping *n* at some viable values such as *n* = 5, 10 and 15, variation of *f*(*m*, *n*) against *m* is shown in Figure 2. Asymptomatic value of *f*(*m*, *n*) is 1/*n* as shown in the figure.

## Minimum values

The minimum value of *f*(*m*, *n*) can be found by differentiating equation (5) w.r.t. *n*, which gives:6
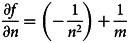


The minimum value of *f*(*m*, *n*) is found for7
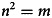


The values of *n* and *m* are related by equation (7). It is interesting to see the pool size can be determined by the division size *m*, which is related to the observed detection rate.

The minimum value for *f*(*m*, *n*) is 2/*n* or 2/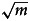, which is reduced to a function of only one variable, say on *m* to give *f*_min_(*m*) as8
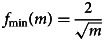


**Fig. 3.**
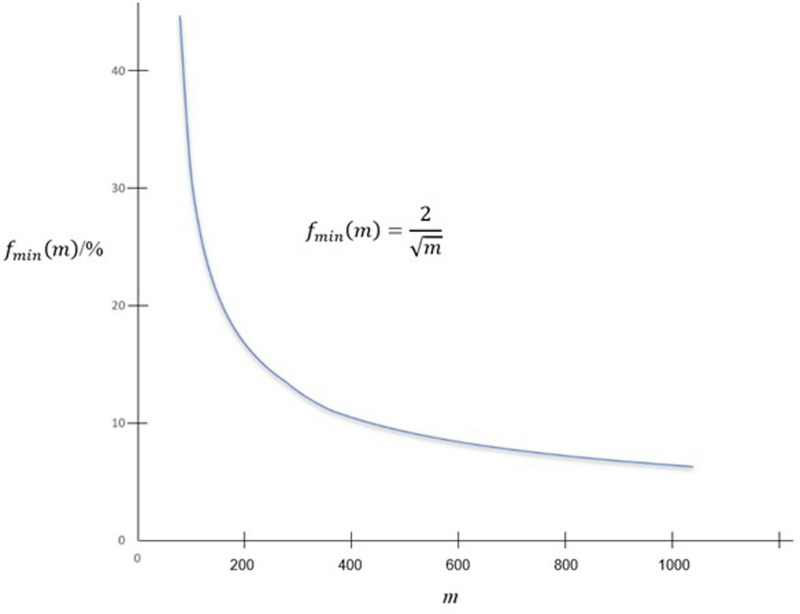
***f***_min_(***m***) against ***m***.

*f*_min_(*m*) is plotted against *m* in Figure 3.

## Discussion on parameters *m* and *n*

Different values of pool size *n* were suggested and used in the literature [15–17]. Pool size of 3−25 was discussed [16] as in Table 1. Other values [25–29] had also been reported. Studies [25] have shown that an individual positive sample can still be detected in pools of up to 32 samples, and possibly even 64 samples provided additional polymerase chain reaction (PCR) amplification cycles. A big pool of 64 samples was used [15, 28]. Value of pooling 10 samples [27] is common, but 30 samples were also used [29].

Values of *m* are different for different risk groups (higher risk for medical professionals or catering services) at different transmission stages. At the beginning stage of virus transmission, the number of confirmed cases is high with patients having obvious symptoms. In testing people in places starting virus transmission [30] at a certain wave, the rate of getting positive results can be high, say one in 100 tests in HKSAR. At a later stage of transmission, the number of confirmed cases decreased to one in 200 tests. The values of *n* in pooling tests can now be derived from observed *m* as discussed in the above section.

For populations in places of lower risk, the value of *m* is larger. For example, 107 AP were identified [31] in testing more than 4 million citizens at Dalian. This gives a low rate of positive results, about one in 40 000, i.e. *m* is 40 000. As the value of *m* was changed, the division number can be changed accordingly at different waves of transmission.

## The management scheme on detection test

A safety management scheme on identifying infected patients with small number of tests is proposed based on the observed detection rate. For a detection rate of one positive result in *m* tests the scheme is as follows:

- Divide the collected population samples *P* into (*P*/*m*) divisions each of *m* people.
- The individual testing samples collected are split into two parts with the first part kept first.
- The second part is grouped with others to give *n* (*n*^2^ = *m*) samples for pool test.
- If the test result is negative, the first part of all *n* samples can be destroyed.
- If there are positive results in the group, the first part of the *n* samples will be tested individually.
- The minimum number of tests per person is only 2/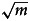 as discussed above.

In a division of 100 people, samples collected can be grouped as 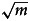 or 10 samples in a pool to test. The number of tests can be reduced to only 20% for individual tests of the population. Reducing the number of tests will consume less test kits, shorten the queuing time for doing the test and reduce the total cost. That means instead of testing 300 000 people a day, the maximum number of people tested can be 1 500 000 a day. A population of 7 million takes less than 5 days to test all citizens.

Testing kits usually have a limit of detection sensitivity, which limits the pool size *n*. Thus the minimum number of tests, or equivalently the maximum value of pool size *n* as obtained by mathematical analysis in this paper is only a theoretical value. The pool size is constrained by sensitivity limit of the test, and *n* could be less than the theoretical value. An initial value of *n* can be estimated first using the curves in Figure 1 under the ideal scenario. Observed detection limit and testing efficiency are then included to tune up the value for optimisation. The maximum value of *m* depends on the maximum value of *n*. At the moment, *n* of 64 is observed to be viable, giving *m* of *n*^2^ as 64 × 64 or 4096. There is a maximum value of *n* so maximum *m* is *n*^2^. Also, there is asymptomatic value of *f* in view of Figures 2 and 3 as *m* increases but with *n* fixed because the number of re-test becomes small as *m* increases.

Governments are always under criticisms not to arrange carrying out detection tests fast. They are also challenged to have management strategy without scientific support. Recommending a pooled testing strategy would be interesting to the governments to conduct interventions in a way that is more resource friendly. Mathematical studies to work out appropriate testing management strategy as above can assist them in convincing the citizens.

Further, issuing health codes [8] for all citizens can ensure that they are safe to travel. But a large number of tests are required regularly because the code is only valid for 3 days at most. For people coming from higher risk areas or groups, the health code only lasts for 1 day, depending on the safety management scheme. The government can implement a scheme for citizens on getting health code for travelling, depending on the maximum number of tests that can be arranged per day.

## Conclusions

In view of the high transmission rate of COVID-19, pooling detection tests is more efficient for a large population. This paper aims at providing an optimal way for testing samples that could release some manpower and potentially speed up the process of testing. The population to be tested is proposed to be divided into divisions based on the recently observed detection rate. The average number of tests per person can be expressed as a function of the observed detection rate and the pool size. The number of people *m* having one positive test result is used in determining the number of divisions in the population and pool size *n*. This scheme can save a lot of time as the number of tests can be greatly reduced. The scheme is therefore very appropriate for identifying AP in a large population, particularly in some places having difficulties in getting test kits.

Health codes are implemented with the code valid for a certain period but it is reduced from 7 days to 3 days in many places. Consequently, a large number of tests are required for millions of people. If the government can handle 0.3 million tests a day, implementing this pooling detection scheme can test 1.5 million samples a day for keeping the health code system viable. This testing strategy is on handling millions of citizens quickly for implementing the health code system which is valid only for a few days. Another important point is to use a smaller number of test kits. This would be useful for government in places with limited resources to implement mass screening for millions of their citizens. The whole idea should be at least not losing any ability in detection, but with acceleration in testing.

This is the first stage of study for implementing universal testing and health code for travelling by using smaller number of test kits. A mass testing scheme proposed to accelerate testing needs support from mathematical principles. Further study should go beyond the mathematics, adding more scenarios or sensitivity analysis to make this scheme more realistic. A more rigorous approach is to compare the pooled method to the unpooled method. The pooled testing should not be significantly inferior to those approved testing. More resources are needed to carry out such studies for comparison. At the moment, based on a summary of pooled testing [14], only a mathematical strategy as mentioned above was reported.

It is important to explore scientific principles on handling virus outbreaks such as developing vaccines and specific medicine. But it takes a long time to get research funding to carry out research and to validate the medicine at various levels of tests, including final clinical tests. Engineering technology hardwares have to be worked out accordingly from selecting scientific principles available and judge whether they are viable. Management has to ensure the hardware systems work as designed and monitored by Authority. Software management needs to control hardware engineering systems. Only with the synergism between science and management could virus outbreaks be under control. Government can only implement regulations effectively with support from citizens. To go one step further, safety can only be achieved on having technology, management and culture.

## Data Availability

Data are available from the authors upon request.

## References

[1] World Health Organization (2020) WHO coronavirus disease (COVID-19) dashboard. Available at https://covid19.who.int/ (Accessed 15 December 2020).

[2] Wong KW, Fung PCW and Chow WK (2020) COVID-19: a physical model. Open Journal of Biophysics 10, 88–95. doi: 10.4236/ojbiphy.2020.102008.

[3] Cheng CH, Chow CL and Chow WK (2020) Trajectories of large respiratory droplets in indoor environment: a simplified approach. Building and Environment 183, 107196. 32836704 10.1016/j.buildenv.2020.107196 PMC7431329

[4] Cheong KH, Wen T and Lai WJ (2020) Relieving cost of epidemic by Parrondo's paradox: a COVID-19 case study. Advanced Science **7**, 2002324. Published online 1 September 2020. doi: 10.1002/advs.202002324. PMC7740105 33344130

[5] Chow WK and Chow CL (2020) A short note on containment scheme against spreading of novel coronavirus COVID-19. Open Journal of Biophysics 10, 84–87. doi: 10.4236/ojbiphy.2020.102007.

[6] Chow WK and Chow CL (2020) A proposed two-stage quarantine containment scheme against spreading of novel coronavirus (SARS-CoV-2). Indoor and Built Environment, Published online first 25 October 2020. doi: 10.1177/1420326X20962154.

[7] Cheong KH and Jones MC (2020) Introducing the 21st century's new four horsemen of the coronapocalypse. BioEssays 42, 2000063. 10.1002/bies.202000063 32227642

[8] Gu M (2020) HK prepares for citywide virus testing. China Daily 2020; 22 August. Available at http://global.chinadaily.com.cn/a/202008/22/WS5f3f8811a310834817261f16.html.

[9] Lai WJ and Cheong KH (2020) Superposition of COVID-19 waves, anticipating a sustained wave, and lessons for the future. BioEssays **42**, 200178. Published online 11 October 2020. doi: 10.1002/bies.202000178. PMC7675615 33040355

[10] Dong D (2020) Why is the cost of the COVID-19 test very low in China? China Daily 2020; 24 April. Available at https://covid-19.chinadaily.com.cn/a/202004/24/WS5ea27e09a310a8b241151694.html.

[11] Ting V, Chan H and Cheung E (2020) Hong Kong third wave: Mainland Chinese personnel to help conduct mass Covid-19 testing in Hong Kong. South China Morning Post 2020; 31 July. Available at https://www.scmp.com/news/hong-kong/health-environment/article/3095444/hong-kong-third-wave-elderly-care-home-resident.

[12] Dorfman R (1943) The detection of defective members of large populations. The Annals of Mathematical Statistics 14, 436–440.

[13] Pikovski A and Bentele K (2020) Pooling of coronavirus tests under unknown prevalence. Epidemiology and Infection 148, e183. 32758313 10.1017/S0950268820001752 PMC7463151

[14] Mallapaty S (2020) The mathematical strategy that could transform coronavirus testing. Nature 583, 504–505. Available at https://www.nature.com/articles/d41586-020-02053-6. 32651561 10.1038/d41586-020-02053-6

[15] Yelin I (2020) Evaluation of COVID-19 RT-qPCR test in multi-sample pools. Clinical Infectious Diseases 71, 2073–2078. doi: 10.1093/cid/ciaa531. 32358960 PMC7197588

[16] Deckert A, Bärnighausen T and Kyei NNA (2020) Simulation of pooled-sample analysis strategies for COVID-19 mass testing. Bulletin of the World Health Organization 98, 590–598. 33012859 10.2471/BLT.20.257188 PMC7463190

[17] Wikipedia. List of countries implementing pool testing strategy against COVID-19. Available at https://en.wikipedia.org/wiki/List_of_countries_implementing_pool_testing_strategy_against_COVID-19 (Accessed 22 August 2020).

[18] Abdalhamid B (2020) Assessment of specimen pooling to conserve SARS CoV-2 testing resources. American Journal of Clinical Pathology 153, 715–718. 32304208 10.1093/ajcp/aqaa064 PMC7188150

[19] Adikari SH (2020) Validation of pooling strategies of clinical COVID-19 samples for more efficient diagnostic testing. Los Alamos National Laboratory (LANL), 23 July 2020.

[20] Adikari SH Investigation of pooling strategies using clinical COVID-19 samples for more efficient diagnostic testing. medRxiv preprint. doi: 10.1101/2020.08.10.20171819.

[21] Park A (2020) How pooled testing for coronavirus could help test more people in less time. Time 2020; 15 July. Available at https://time.com/5867102/pooled-testing-coronavirus/.

[22] Cheung E (2020) Hong Kong third wave: universal Covid-19 testing tougher than it sounds, say health experts, who urge targeted screenings, continued social distancing. South China Morning Post 2020; 5 August. Available at https://www.scmp.com/news/hong-kong/health-environment/article/3095987/hong-kong-third-wave-universal-covid-19-testing.

[23] Sinnott-Armstrong N, Klein DL and Hickey B (2020) Evaluation of group testing for SARS-CoV-2 RNA. 25 March 2020, medRxiv preprint. doi: 10.1101/2020.03.27.20043968.

[24] Ben-Ami R (2020) Large-scale implementation of pooled RNA extraction and RT-PCR for SARS-CoV-2 detection. Clinical Microbiology and Infection 26, 1248–1253. 32585353 10.1016/j.cmi.2020.06.009 PMC7308776

[25] Sunjaya AF and Sunjaya AP (2020) Pooled testing for expanding Covid-19 mass surveillance. Disaster Medicine and Public Health Preparedness 14, E42–E43. doi: 10.1017/dmp.2020.246. PMC7443553 32660684

[26] Chow WK and Chow CL (2020) Comment on ‘Sunjaya AF, Sunjaya AP. Pooled testing for expanding COVID-19 mass surveillance. Disaster Medicine and Public Health Preparedness. 14 July 2020’. Disaster Medicine and Public Health Preparedness, Published online 19 November 2020. Available at 10.1017/dmp.2020.454. PMC7443553 32660684

[27] Elsevier (2020) Pooling strategy in the wake of the COVID-19 pandemic: A solution for mass population screening of SARS-CoV-2, 30 July 2020. Available at https://www.elsevier.com/about/press-releases/research-and-journals/pooling-strategy-in-the-wake-of-the-covid-19-pandemic.

[28] Majid F, Omer SB and Khwaja AI (2020) Optimising SARS-CoV-2 pooled testing for low-resource settings. The Lancet Microbe. Comment. 1, e101–e102. Published Online June 8, 2020. doi: 10.1016/S2666-5247(20)30056-2. 32835337 PMC7279755

[29] Mishra B (2020) Challenges and issues of SARS-CoV-2 pool testing. The Lancet Infectious Diseases, Correspondence **23**, 1233. Published Online July 14, 2020. doi: 10.1016/S1473-3099(20)30463-1. PMC7831385 32679083

[30] Long DZ (2020) Coronavirus third wave: group testing can address Hong Kong's limited capacity. South China Morning Post 2020; 17 July. Available at https://www.scmp.com/comment/opinion/article/3093464/coronavirus-third-wave-group-testing-can-address-hong-kongs-limited.

[31] Tellerrreport.com (2020) Nucleic acid sampling in Dalian's main urban area has completed a total of 4.488 million nucleic acid tests. 1 August 2020. Available at https://www.tellerreport.com/life/2020-08-01-nucleic-acid-sampling-in-dalian-s-main-urban-area-has-completed-a-total-of-4-488-million-nucleic-acid-tests.B1VN6hOGbv.html.

